# Seasonal and Temporal Variation in Release of Antibiotics in Hospital Wastewater: Estimation Using Continuous and Grab Sampling

**DOI:** 10.1371/journal.pone.0068715

**Published:** 2013-07-08

**Authors:** Vishal Diwan, Cecilia Stålsby Lundborg, Ashok J. Tamhankar

**Affiliations:** 1 Global Health (IHCAR), Department of Public Health Sciences, Karolinska Institutet, Tomtebodavägen, Stockholm, Sweden; 2 Department of Public Health & Environment, R.D. Gardi Medical College, Agar Road, Ujjain, India; 3 Indian Initiative for Management of Antibiotic Resistance (IIMAR), Department of Environmental Medicine, R.D. Gardi Medical College, Agar Road, Ujjain, India; Kenya Medical Research Institute - Wellcome Trust Research Programme, Kenya

## Abstract

The presence of antibiotics in the environment and their subsequent impact on resistance development has raised concerns globally. Hospitals are a major source of antibiotics released into the environment. To reduce these residues, research to improve knowledge of the dynamics of antibiotic release from hospitals is essential. Therefore, we undertook a study to estimate seasonal and temporal variation in antibiotic release from two hospitals in India over a period of two years. For this, 6 sampling sessions of 24 hours each were conducted in the three prominent seasons of India, at all wastewater outlets of the two hospitals, using continuous and grab sampling methods. An in-house wastewater sampler was designed for continuous sampling. Eight antibiotics from four major antibiotic groups were selected for the study. To understand the temporal pattern of antibiotic release, each of the 24-hour sessions were divided in three sub-sampling sessions of 8 hours each. Solid phase extraction followed by liquid chromatography/tandem mass spectrometry (LC-MS/MS) was used to determine the antibiotic residues. Six of the eight antibiotics studied were detected in the wastewater samples. Both continuous and grab sampling methods indicated that the highest quantities of fluoroquinolones were released in winter followed by the rainy season and the summer. No temporal pattern in antibiotic release was detected. In general, in a common timeframe, continuous sampling showed less concentration of antibiotics in wastewater as compared to grab sampling. It is suggested that continuous sampling should be the method of choice as grab sampling gives erroneous results, it being indicative of the quantities of antibiotics present in wastewater only at the time of sampling. Based on our studies, calculations indicate that from hospitals in India, an estimated 89, 1 and 25 ng/L/day of fluroquinolones, metronidazole and sulfamethoxazole respectively, might be getting released into the environment per 100 hospital beds.

## Introduction

One of the contributory factors implicated for the development of antibiotic resistance, is the exposure of bacteria to antibiotics in the environment, particularly the aquatic environment [1]. Significant quantities of antibiotics in unchanged form or as active metabolites enter into the aquatic environment through hospital wastewater, pharmaceutical plant effluents, disposal of unused antibiotics from residential and commercial establishments, animal feeding operations and aquaculture [2], [3], [4], [5], [6]. Due to high analytical costs, occurrence of antibiotic residues in the environment is mostly reported from high-income countries, with relatively few reports from low- and middle-income countries [2], [3], [6], [7], [8], [9], [10], [11], [12]. Knowledge generation in this aspect is however more important in low- and middle-income countries, as wastewater in these countries undergoes little or no treatment before entering fresh water sources and therefore contingent risks need to be evaluated. Moreover, estimates from India have shown an increased use of antibiotics, resulting in an increased release into the environment [13].

Therefore, we undertook a two-year study to estimate antibiotic residues in wastewater of two representative hospitals in India, with the aim of understanding seasonal and temporal variation in antibiotic release. Sampling was done using continuous and grab sampling methods to compare antibiotic release estimates given by the two methods. The long-term aim is to introduce interventions to reduce/remove antibiotic residues from hospital wastewater.

## Materials and Methods

### Study Setting

The study was conducted in the central Indian province, Madhya Pradesh (MP). The sampling sites were two hospitals in Ujjain district. ‘Hospital 1’ is a non-teaching hospital with 350 patient beds located in Ujjain city catering mainly to city population, while ‘Hospital 2’, is a 570-bed teaching hospital attached to a medical college, located in a rural area 6 km from the city and catering predominantly to the rural population. Both hospitals have all major medical specialties.

### Sampling Protocol

To understand the seasonal variation in antibiotic residues in hospital wastewater, the study was conducted during the three prominent seasons of India, i.e. summer, rainy season and winter. During these three seasons, weather conditions in the study setting remain as follows [14]. The summer months, March to June, have monthly average maximum temperatures respectively of 35, 39, 40, and 36°C, with maximum temperature reaching up to 45°C on many days. During this period, the monthly average minimum temperatures are respectively 17, 21, 25, and 24°C. The monsoon weather system, which causes rain in India, arrives in this area in the later part of June and the months of June until September receive average rainfall respectively of 144, 274, 124 and 146 mm. The monthly average maximum temperature during July to September is respectively 30, 29, and 31°C and the monthly average minimum temperature is respectively 23, 22, and 21°C. Winter months are from November to February and the monthly average maximum temperatures in the winter months are 31, 28, 27, and 30°C respectively, the monthly average minimum temperatures being 14, 17, 10, and 12°C. On certain days during the winter months, minimum temperatures of 3°C also get recorded in the night. The month of October, which intervenes between rainy season and winter, has average maximum temperature of 33°C and average minimum temperature of 18°C, the average rainfall during this month being 30 mm.

Summer sampling was done during the second week of May 2008 and the third week of May 2009. Rainy season sampling was done during the last week of July 2008 and the first week of August 2009. Winter sampling was done during the last week of January 2009 and the first week of February 2010. In total, six sampling sessions of 24 hours each were conducted in each hospital during the study (one sampling session in each hospital for each of the three seasons during each year). Furthermore, to understand the temporal pattern of antibiotic release from the hospitals, each of these 24-hour sessions was divided into three sub-sampling sessions of 8 hours each. The three sub-sampling sessions were, 0900–1700, 1700–0100 and 0100–0900 hours. The first sub-sampling session (0900–1700 hours) represented the hottest 8 hours of the day, when sunshine is relatively intense. The second sub-sampling session (1700–0100 hours) represented the cooling period and the third sub-sampling session (0100–0900 hours) represented the relatively cooler period of the 24 hours. This sequence of sampling was also convenient from the point of view of logistics of working.

### Sampling Procedure

Wastewater sampling was done at all the wastewater outlets of the two hospital buildings at the points where water came out of the hospitals after use; four outlets in Hospital 1, and 6 in Hospital 2. Wastewater from each of the hospitals’ outlets was collected in a small wastewater chamber (2′×2′×2′) before it exited the hospitals’ premises. The wastewater in these chambers was sampled using (i) continuous, and (ii) grab methods.

### Continuous Sampling

**Figure 1:**
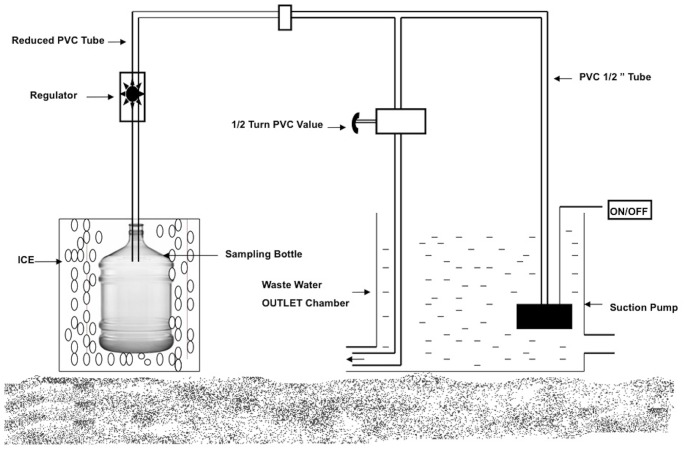
Representative diagram of the continuous water sampler.

An in-house wastewater sampler was designed for continuous sampling, which consisted of an electrically-operated submersible pump (230V, 50 Hz, 16W), a polyvinyl chloride (PVC) tube (1/2 inch dia.) with flow regulator, a half turn PVC valve, a PVC Tee (T) joint (1/2 inch dia.), electric (on/off) switch and an indicator lamp to show power on/off. A representative diagram of the wastewater sampler’s assembly is given in Figure 1. Each of the hospitals’ wastewater chambers had an independent wastewater sampling assembly. Wastewater was collected in glass bottles that were earlier washed with dilute soap water, followed by double distilled water and finally sterilized. All PVC components of the sampler were washed internally and externally, first with diluted soap water and then with double-distilled water. Before use, a mock run of the assembly was conducted using double-distilled water. During the mock run, the half turn valve and flow regulator were adjusted so that one-liter of water could be collected in the sampling bottle in one hour. For the purpose of sampling, the assembly was so arranged that the suction pump remained submerged in the wastewater chamber and the sample collection bottle remained at ground level near the chamber, inside a PVC box filled with ice. When power was switched on, the pump started running and water flowed through the assembly and fell into the sampling bottle. Water in excess of the required sampling quantity (1L/hour), if any, exited through the other opening of the T and was carried away into the drainage system.

On each sampling day, the 24 hours sampling session consisted of three consecutive continuous sub-sampling sessions of 8 hours each (0900–1700, 1700–0100 and 0100–0900 hours), in both the hospitals. At the end of every 8-hour session, all wastewater samples collected from all the outlets of each hospital were mixed together to make a composite sample representing that 8-hour duration for that hospital. From this sample, two liters of wastewater was sent for chemical analysis.

### Grab Sampling

Grab sampling consisted of taking wastewater samples instantly at one point in time. In this method, in each sampling session, a one-time separate grab sample (two liters each) was collected from all the wastewater chambers of both the hospitals, once in 8 hours. In ‘Hospital 1’, grab samples were collected at 1300, 2100 and 0500 hours, while in ‘Hospital 2’, they were collected at 1000, 1800 and 0200 hours. From a feasibility point-of-view, the sampling times and days for the two hospitals had to be kept different, but it was done on consecutive days. All samples from all the wastewater chambers of a hospital, taken at one time e.g. say at 1300 or 1000 hours were mixed to make one grab sample representing that sampling time. From this mixture of grab samples, a two-liter sample was sent for chemical analysis. In all, three grab samples were made from each hospital during each 24-hour sampling session.

### Sampling of Incoming Water

Both the hospitals received water from municipal as well as ground water sources. The water was received once daily in a water tank, located in the hospital area. Samples (2 L) representing the incoming water of each hospital were collected from the water tank at 0800 hours on the day of sampling as a one-time grab sample in each season and in each hospital. This was done to determine if the incoming water was already contaminated with antibiotics and also to ascertain that the quantities of antibiotics detected in the hospital wastewater represented the actual release by the hospitals.

### Sample Storage and Handling

To prevent degradation of the antibiotics, all samples were placed in screw-capped amber bottles wrapped in silver foil. The samples were stored at <4°C immediately after collection until they reached the analytical laboratory (within 24 hours). Analysis was performed at the laboratories of Shriram Institute for Industrial Research, New Delhi, where the samples remained at −20°C until analyses. Details of sample storage and handling methods are described in Diwan *et al* 2010 [12].

### Antibiotic Selection

Eight antibiotics from four major antibiotic groups - cephalosporins (ceftriaxone and cefoperazone), fluoroquinolones (ofloxacin, ciprofloxacin, norfloxacin, levofloxacin), sulfonamides (sulfamethoxazole) and imidazoles (metronidazole) - were selected for analyses. The selection was based on, (i) the prescription pattern in the inpatient wards of the hospitals, (ii) antibiotic residues found in the same setting in our previous study [12], (iii) the degree of antibiotic metabolism by the human body, (iv) environmental stability, and (v) the known and suspected environmental impact of an antibiotic [15].

### Analyses

The details of the method of analyses can be found in Diwan *et al* 2010 [12]. In brief, the determination of antibiotic residues in hospital-associated waters was done after selectively isolating the analytes from the matrix using solid phase extraction followed by LC-MS/MS (Waters 2695 series Alliance quaternary liquid chromatography system, Waters, USA, with a triple quadruple mass spectrometer, Quatro-micro API, Micromass, UK, equipped with electro-spray interface and Masslynx 4.1 software (Micromass, UK) for data acquisition and processing). The method was validated as per the International Conference on Harmonisation (ICH) and Eurachem guidelines [16], [17]. The limit of quantification (LOQ) and limit of detection (LOD) in ng/L during LC-MS/MS analysis were respectively, ceftriaxone - 2.5/5.0, ofloxacin - 0.01/0.025, ciprofloxacin - 0.01/0.025, norfloxacin - 0.01/0.025, levofloxacin - 0.01/0.025, metronidazole - 0.01/0.01 and sulfamethoxazole - 0.01/5.0.

### Ethical Approval

Both the hospitals and R.D. Gardi Medical College are interlinked institutions and the study was approved for both the hospitals by the ethics committee of R.D. Gardi Medical College, Ujjain (No. 41/2007).

## Results

**Figure 2:**
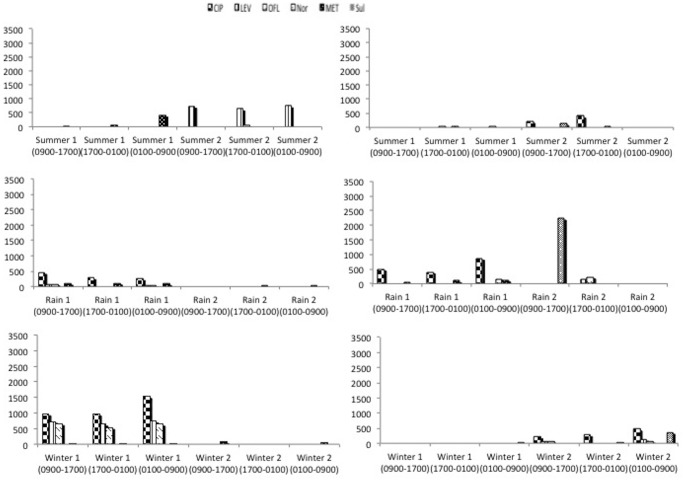
Seasonal and temporal variation in antibiotic residues (ng/L) in the wastewater of the two hospitals by continuous sampling.

**Figure 3:**
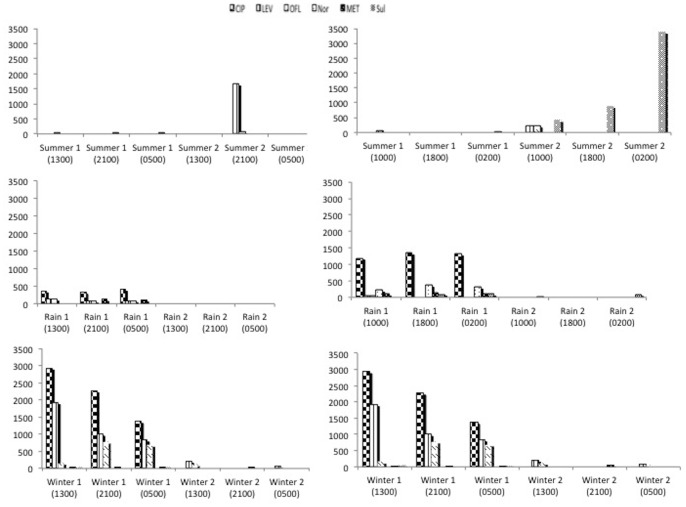
Seasonal and temporal variation in antibiotic residues (ng/L) in the wastewater of the two hospitals by grab sampling.

The seasonal and temporal variation in antibiotic residues in the wastewater of the two hospitals obtained by continuous and grab sampling methods, is presented respectively in figures 2 and 3 In general, 6 of the 8 antibiotics studied were detected in the wastewater samples. These antibiotics belonged to three groups - fluoroquinolones (ciprofloxacin, levofloxacin, ofloxacin, norfloxacin), imidazoles (metronidazole) and sulphonamides (sulfamethoxazole). Antibiotics from the cephalosporin group (ceftriaxone and cefeperazone) were not detected in samples from any of the hospitals, whereas norfloxacin and sulfamethoxazole were never detected in the wastewater of ‘Hospital 1’. In general, in a common time frame, in most cases, continuous sampling showed less antibiotic concentration in wastewater compared to grab sampling. For incoming water, no antibiotics were detected in any of the samples from the two hospitals. The seasonal and temporal concentration variation in antibiotic residues in the wastewater of the two hospitals by continuous and grab sampling method are available as additional material (Tables S1 and S2 respectively).

## Discussion

To our knowledge, this is the only study worldwide which has attempted to quantify antibiotic residues in hospital wastewater using concurrent continuous and grab sampling methods. Furthermore, the sampling in our study was done at locations closest to the hospitals’ wastewater outlets, whereas in most of the earlier published studies, sampling was done at the inlet and outlet points of wastewater treatment sites of hospitals, by the grab method [11], [18], [19].

No antibiotic residues were detected in the incoming water, indicating that the entire quantities of antibiotic residues detected in the hospital waste water were actually released by the hospitals**.** The water released from the hospitals contained antibiotics belonging to the fluoroquinolone, imidazole and sulphonamide groups. Although cephalosporins are prescribed in significant quantities in the hospitals [12], they were not detected in any of the wastewater samples. This follows observations in other studies, where cephalosporins were not detected in hospital wastewater and the reason cited is the easy degradation of the ß-lactam ring, its high metabolic rate and the process of decarboxylation [2].

Variation in the detection of antibiotic residues in the wastewater of the two hospitals was observed, between seasons, between sampling times and between the two years of sampling. Detecting variation in antibiotic residues in the wastewater of any two hospitals is always likely because of variation in patient admission with different maladies, preference and type of antibiotics prescribed and administration, variation in patient behavior and physicochemical behavior of antibiotics [20], [21]. The predicted no effect concentration (PNEC) for total fluoroquinolones in wastewater for *Pseudomnas putida* is 8,000 ng/L and for fish, daphnia and algae it is 3,000 ng/L [22]. In the present study, the reported fluoroquinolones concentration in hospital wastewater was much lower than the PNEC reported elsewhere. Although data available from other countries with respect to antibiotic residues in hospital wastewater has been obtained using the ‘grab’ sampling method [11], [18], it was observed that, comparatively, the antibiotic release from the hospitals in this study was much less.

### Seasonal Variation in Antibiotic Residues

In the two hospitals, the number and concentration of antibiotics detected in wastewater varied from one season to another. There was also no similarity in the occurrence of antibiotic residues in the same season of the two years. When the continuous sampling method was used, concentrations of antibiotics detected in winter and rainy season were more than four times higher than in summer (Table 1). For the same time frame, the grab sampling method showed an increasing trend from summer to the rainy season to winter. In our experiments, levels of fluoroquinolone residues were highest during winter. Results from a study in Delhi, India, encompassing private retail pharmacies, public healthcare facilities and private clinics, showed a slightly higher consumption of some antibiotics in winter and slightly higher consumption of fluoroquinolones during the rainy season [21]. Similar differences may exist in our setting which, to some extent, might explain the seasonal trend of antibiotic residues found in our study. Also, in other settings, when water other than hospital wastewater was analyzed for antibiotic residues, it was observed that in winter, antibiotic levels were higher when compared to other seasons [23], [24]. Similarly, in river water, lower antibiotic residues have been reported in summer [25]. Seasonal variation in antibiotic residues was also observed in hospital effluents in Portugal, with antibiotic levels higher in spring than in autumn [26]. Although we sampled water as it exited the hospital building, the variable role of natural factors occurring in various seasons cannot be ruled out. Possible reasons for fewer antibiotics detected in wastewater in summer could be, higher activity of microorganisms, intense sunlight and high temperatures during summer, which could have caused more biodegradation and photodegradation [27]. A perusal of the information given earlier regarding weather conditions shows that the temperatures during summer in our study setting are nearly double than that observed in winter. In general, taking into consideration the variable weather conditions mentioned earlier in sampling protocol for various seasons in the study setting, the observed variation in antibiotics residues in wastewater during various seasons can be expected.

**Table 1:** Concentration of antibiotic released/day/hospital (ng/L).

	CIP		LEV		OFL		NOR		FQ		MET		SUL		TOTAL	
	CS	GS	CS	GS	CS	GS	CS	GS	CS	GS	CS	GS	CS	GS	CS	GS
Summer	155.5	–	–	472	35	96	–	–	191	568	131	18	34	1174	355	1761
Rains	694	1239	66	88	90	85	40	225	891	1638	145	143	560	76	1596	1858
Winter	245	1836	578	1078	495	475	–	–	1318	3389	36	18	106	355	1460	3763
Total	1095	3076	644	1638	620	656	40	225	2400	5595	312	179	700	1605	3412	7381
Average (Total/3)	365	1025	214	546	206	218	13	75	800	1865	104	59.95	233	535	1137	2461

CIP: Ciprofloxacin, LEV: Levofloxacin, OFL: Ofloxacin, NOR: Norfloxacin, FQ: fluroquinolones, MET: Metronidazole, SUL: sulfamethoxazole,

CS: Continuous sampling, GS: Grab Sampling.

### Temporal Variation in Antibiotic Residues

In general, very little information is available on the temporal variation in antibiotic residues in the aquatic environment, particularly in hospital wastewater. Conducting studies on municipal wastewater in Sweden using grab sampling method and taking samples at two-hour intervals only between 0900 to 2200 hours (not all the 24 hours), Lindberg *et al* reported a higher concentration of ciprofloxacin at 1300 and 1500 hours and of ofloxacin at 1300 and 1700 hours [19]. Our results are not directly comparable with Lindberg *et al* as we conducted our studies in tropical conditions in India, took samples in three seasons during three time frames over 24 hours and also employed two sampling methods. However, if a comparison had to be made under our conditions, the ambient temperatures in winter appear somewhat similar to summer temperatures in Sweden and during this season, using the grab sampling method (as used by Lindberg *et al* [19]), we found ciprofloxacin in highest quantities at 1300 hours during 2009 and ofloxacin in nearly similar amounts at 2100 and 0500 hours in winter 2010 at ‘Hospital 1’. In the other hospital, ciprofloxacin and ofloxacin were detected in highest quantities at 0200 hours. Results of continuous sampling showed highest quantities of ciprofloxacin during winter between 0100 to 0900 hours in both the hospitals. Ofloxacin was detected in nearly similar quantities in the same timeframe. Only once did ciprofloxacin show results similar to Lindberg *et al* [19]. The water temperature during our sampling in winter was between 9–24°C. When grab sampling was done at three hour intervals over a 24-hour period in a hospital in Hanoi (Vietnam), with water temperatures ranging between 15–20°C, highest concentration of ciprofloxacin was detected at 2300 hours (∼ 45 µg/L) and that of norfloxacin at 1700 hours (∼ 9 µg/L) [11].

It is suggested that temporal variation in antibiotic concentration in wastewater might arise due to variation in antibiotic administration during the course of the day and various pharmacokinetic factors such as metabolism, half-life and excretion, and environmental factors like flow and temperature [24], [28]. In general, in spite of conducting continuous sampling over a 24-hour time frame in each season over two years, no specific temporal pattern in detection of antibiotic residues was observed in our studies.

### Comparison of Continuous and Grab Sampling

The two different sampling methods we experimented on, indicated the release of different numbers and concentrations of antibiotics into hospital wastewater for the same time frame and even for the same hospital. In both the hospitals, the concentration of antibiotic residues detected by grab sampling was mostly on the higher side compared to continuous sampling. For a given time frame however, grab samples only give a ‘snap-shot’ picture, and these results are not representative of a whole timeframe. The continuous sampling method on the other hand, samples the wastewater continuously during the whole period and gives an estimation of the antibiotic residues for the whole timeframe in question. Low antibiotic levels observed in samples obtained by the continuous sampling method may be explained by a dilution effect; in continuous sampling, even when there is no antibiotic residue present in the wastewater, the sample is taken regardless and gets mixed with the total sample.

To our knowledge, simultaneous sampling of hospital wastewater by grab and continuous methods has not been done earlier. It is evident from our studies however, that grab sampling gives an erroneous picture of the antibiotic residues present in wastewater, if the wastewater in question is a dynamic system. Grab sampling may be useful to some extent, to study antibiotic residues in stagnant water or when information on antibiotic residues is wanted for a particular point in time. For dynamic systems of water, we suggest use of continuous sampling over a time period, to obtain a more realistic picture of the status of antibiotic residues.

### Projections

If an attempt to generalize the quantities of antibiotics released into the environment from hospitals is made using information from Table 1, it can be extrapolated that for every 100 hospital beds (the two studied hospitals have a total of 920 beds) existing in similar conditions in India, the amount of fluoroquinolones, metronidazole and sulfamethoxazole released in hospital wastewater will be ∼89 ng/L/day, ∼11 ng/L/day and ∼25 ng/L/day, respectively, as indicated by the continuous sampling method is employed. The grab sampling method indicates these figures to be ∼207, ∼6 and ∼60 ng/L/day, respectively. For reasons mentioned earlier, we feel that the figures obtained by the continuous sampling method will be more reliable and that since we did sampling for two years in two hospitals for all the three seasons, over 24 hours, the average figures obtained in our results may be applicable to similar settings in many parts of the world. To strengthen our argument, one may add here that in a surveillance study of antibiotic consumption conducted between November 2007 and February 2009 using the “focus of infection” approach in the same two hospitals, (a period partly coinciding with our studies), which included 6026 admitted patients, it was found that antibiotics were prescribed to 92% of the patients and fluoroquinolones and cephalosporins were the highest prescribed antibiotics [29]. In our study, fluoroquinolones were detected in highest quantities in hospital wastewater, but not cephalosporins. It has already been explained that cephalosporins are easily degraded in the environment. Thus, the results of the antibiotic residue analysis coincide with the antibiotic prescription patterns in these two hospitals. If these calculations are extended further, we can derive that, per hospital bed, ∼0.9, ∼0.12 and ∼0.26 ng/L/day of ciprofloxacin, metronidazole and sulfamethoxazole respectively, might be getting released into the environment. It will not be too ambitious to say that, these calculations also have some bearing on the community’s antibiotic use and its release into the surrounding environment.

Gullberg *et al* showed that selection of resistant bacteria occurs at antibiotic concentrations several hundred-fold below the minimal inhibitory concentration (MIC) and de novo mutants get selected very rapidly at sub-MIC antibiotic concentrations and take over a susceptible population [30]. We have already demonstrated, in parallel studies conducted using samples from the same hospital wastewater, that this hospital wastewater contains *E. coli* populations that have constituents that are ESBL-positive and are carrying resistance genes such as *bla*_CTX-M_, *bla*_TEM,_ *bla*_SHV_ and *qnrA, qnrB*, *qnrS, aac (6′)-Ib-cr*, *qepA* [31]. Taken together, our results from these studies, suggest that resistance may be evolving all over the world in hospital wastewaters, where our results show that low antibiotic concentrations may be existing all the time, that are important for enrichment and maintenance of resistance in bacterial populations.

### Conclusions and Implications

The aim of this study was to understand the seasonal and temporal dynamics of antibiotic residue levels in hospital-associated waters from the point-of-view of administering interventions to reduce/remove antibiotic residues from the wastewater. It appears that such interventions cannot be time and season specific, as antibiotics are released in hospital wastewater continuously, daily and all year round, with not much seasonal or temporal variation. Further based on our studies, calculations indicate that from hospitals in India, an estimated 89, 1 and 25 ng/L/day of fluroquinolones, metronidazole and sulfamethoxazole respectively, might be getting released into the environment per 100 hospital beds. More studies are needed in this area to confirm present findings. We think that if knowledge regarding realistic levels of antibiotic residues in hospital wastewater is to be obtained for the planning of interventions, then the continuous sampling method should be the method of choice.

## Supporting Information

Table S1 **Seasonal and temporal variation in antibiotic residues (ng/L) in the wastewater of the two hospitals by continuous sampling.**
(DOC)

Table S2 **Seasonal and temporal variations in antibiotic residues (ng/L) in the wastewater of the two hospitals by grab sampling.**
(DOC)
