# Supplementary material for: Seasonal and Temporal Variation in Release of Antibiotics in Hospital Wastewater: Estimation Using Continuous and Grab Sampling
Source: PLoS One. 2013 Jul 8;8(7):e68715. doi: 10.1371/journal.pone.0068715 (PMC3704537; doi:10.1371/journal.pone.0068715)
Supplement: Table S1 — Seasonal and temporal variation in antibiotic residues (ng/L) in the wastewater of the two hospitals by continuous sampling. (DOC) [file pone.0068715.s001.doc]

Table: S1 Seasonal and temporal variation in antibiotic residues (ng/L) in the wastewater of the two hospitals by continuous sampling

|  |  | **CIP** | | **LEV** | | **OFL** | | **NOR** | | | **MET** | | **SUL** | | **CEFT** | | **CEFO** | |
| --- | --- | --- | --- | --- | --- | --- | --- | --- | --- | --- | --- | --- | --- | --- | --- | --- | --- | --- |
| **Seasons** | **Time (hr)** | **H1** | **H2** | **H1** | **H2** | **H1** | **H2** | | **H1** | **H2** | **H1** | **H2** | **H1** | **H2** | **H1** | **H2** | **H1** | **H2** |
| Summer 1 | 0900-1700 | - | - | - | - | - | - | | - | - | 33.2 | - | - | - | - | - | - | - |
|  | 1700-0100 | - | - | - | - | - | 53 | | - | - | 50 | 9.2 | - | - | - | - | - | - |
|  | 0100-0900 | - | - | - | - | - | 26 | | - | - | 417 | - | - | - | - | - | - | - |
| Summer 1 | 0900-1700 | - | 214 | 730 | - | - | - | | - | - | - | - | - | 136 | - | - | - | - |
|  | 1700-0100 | - | 408 | 640 | - | 63 | - | | - | - | - | 13 | - | - | - | - | - | - |
|  | 0100-0900 | - | - | 750 | - | - | - | | - | - | - | - | - | - | - | - | - | - |
| Rain 1 | 0900-1700 | 463 | 500 | 64 | - | 83 | - | | - | - | 108 | 13.7 | - | - | - | - | - | - |
|  | 1700-0100 | 291 | 398 | - | - | - | - | | - | - | 115 | 104 | - | - | - | - | - | - |
|  | 0100-0900 | 259 | 868 | 51 | - | 48 | - | | - | 160 | 99 | 127 | - | - | - | - | - | - |
| Rain 1 | 0900-1700 | - | - | - | - | - | - | | - | - | - | - | - | 2240 | - | - | - | - |
|  | 1700-0100 | - | - | - | 150 | - | 230 | | - | - | 6.7 | - | - | - | - | - | - | - |
|  | 0100-0900 | - | - | - | - | - | - | | - | - | 7 | - | - | - | - | - | - | - |
| Winter 1 | 0900-1700 | 976 | - | 730 | - | 660 | - | | - | - | 9.6 | - | - | - | - | - | - | - |
|  | 1700-0100 | 972 | - | 640 | - | 537 | - | | - | - | 8 | - | - | - | - | - | - | - |
|  | 0100-0900 | 1530 | - | 750 | - | 640 | - | | - | - | 13 | - | - | 21 | - | - | - | - |
| Winter 2 | 0900-1700 | - | 218 | - | 61 | - | 71 | | - | - | 76 | - | - | - | - | - | - | - |
|  | 1700-0100 | - | 288 | - | - | - | - | | - | - | - | - | - | 38 | - | - | - | - |
|  | 0100-0900 | - | 474 | - | 132 | - | 72 | | - | - | 40 | - | - | 365 | - | - | - | - |

H1: Hospital 1, Hospital 2

CIP: Ciprofloxacin, LEV: Levofloxacin, OFL: Ofloxacin , NOR:Norfloxacin, MET:Metronidazole, SUL:Sulphamethaxazole, CEFT:Ceftriaxone, CEFO: Cefoperazone

(-) : Below Detection Level
