# Supplementary material for: Seasonal and Temporal Variation in Release of Antibiotics in Hospital Wastewater: Estimation Using Continuous and Grab Sampling
Source: PLoS One. 2013 Jul 8;8(7):e68715. doi: 10.1371/journal.pone.0068715 (PMC3704537; doi:10.1371/journal.pone.0068715)
Supplement: Table S2 — Seasonal and temporal variations in antibiotic residues (ng/L) in the wastewater of the two hospitals by grab sampling. (DOC) [file pone.0068715.s002.doc]

**Table S2:** Seasonal and temporal variations in antibiotic residues (ng/L) in the wastewater of the two hospitals by grab sampling

|  |  | **CIP** | | **LEV** | | **OFL** | | **NOR** | | **MET** | | **SUL** | | **CEFT** | | **CEFO** | |
| --- | --- | --- | --- | --- | --- | --- | --- | --- | --- | --- | --- | --- | --- | --- | --- | --- | --- |
| **Seasons** | **Time (hr)** | H1 | H2 | H1 | H2 | H1 | H2 | H1 | H2 | H1 | H2 | H1 | H2 | H1 | H2 | H1 | H2 |
| Summer 1 | **1^st^** | - | - | - | - | 34.6 | 57.1 | - | - | - | - | - | - | - | - | - | - |
|  | **2^nd^** | - | - | - | - | - | - | - | - | 29.6 | - | - | - | - | - | - | - |
|  | **3^rd^** | - | - | - | - | - | - | - | - | 31.7 | 11 | - | - | - | - | - | - |
| Summer 2 | **1^st^** | - | - | - | 229 | - | 227 | - | - | - | - | - | 417 | - | - | - | - |
|  | **2^nd^** | - | - | 1660 | - | 66 | - | - | - | - | - | - | 890 | - | - | - | - |
|  | **3^rd^** | - | - | - | - | - | - | - | - | - | - | - | 3390 | - | - | - | - |
| Rain 1 | **1^st^** | 365 | 1190 | 143 | 46 | 132 | 50 | - | 220 | - | 101 | - | - | - | - | - | - |
|  | **2^nd^** | 328 | 1350 | 81 | - | 71 | - | - | 370 | 119 | 130 | - | 92.9 | - | - | - | - |
|  | **3^rd^** | 406 | 1320 | 84 | - | 86 | - | - | 310 | 111 | 113 | - | 96.4 | - | - | - | - |
| Rain 2 | **1^st^** | - | - | - | - | - | - | - | - | - | - | - | 29 | - | - | - | - |
|  | **2^nd^** | - | - | - | - | - | - | - | - | - | - | - | - | - | - | - | - |
|  | **3^rd^** | - | - | - | - | - | - | - | - | - | - | - | 86 | - | - | - | - |
| Winter 1 | **1^st^** | 2930 | - | 1920 | - | 162 | - | - | - | 6 | - | 50 | - | - | - | - | - |
|  | **2^nd^** | 2270 | - | 1000 | - | 781 | - | - | - | 13.7 | - | - | - | - | - | - | - |
|  | **3^rd^** | 1370 | - | 830 | - | 690 | - | - | - | 7 | - | 22.7 | - | - | - | - | - |
| Winter 2 | **1^st^** | - | 184 | 206 | 84 | 123 | - | - | - | - | - | - | 918 | - | - | - | - |
|  | **2^nd^** | - | 229 | - | - | - | - | - | - | 46 | - | - | - | - | - | - | - |
|  | **3^rd^** | - | 362 | 76 | 196 | 40 | 105 | - | - | - | - | - | 431 | - | - | - | - |

H1: Hospital 1, Hospital 2

CIP: Ciprofloxacin, LEV: Levofloxacin, OFL: Ofloxacin , NOR:Norfloxacin, MET:Metronidazole, SUL:Sulphamethaxazole, CEFT:Ceftriaxone, CEFO: Cefoperazone

For H1: 1^st^  : Sampling at 1300 Hours, 2^nd^ : Sampling at 2100 Hours, 3^rd^ : Sampling at 0500 Hours

For H2 : 1^st^ : Sampling at 1000 Hours, 2^nd^ : Sampling at 1800 Hours, 3^rd^ : Sampling at 0200 Hours

(-) : Below Detection Level
